# Adolescents’ Use of Nicotine-Free and Nicotine E-Cigarettes: A Longitudinal Study of Vaping Transitions and Vaper Characteristics

**DOI:** 10.1093/ntr/ntab192

**Published:** 2021-09-21

**Authors:** Rikke Tokle, Geir Scott Brunborg, Tord Finne Vedøy

**Affiliations:** 1 Norwegian Social Research, Oslo Metropolitan University, Oslo, Norway; 2 Department of Alcohol, Tobacco and Drugs, Norwegian Institute of Public Health, Oslo, Norway

## Abstract

**Introduction:**

Although adolescents’ nicotine addiction from e-cigarettes is a concern, few studies differentiate between vaping with and without nicotine. This study examines the prevalence of nicotine and nicotine-free vaping, maps transitions between vaping behaviors, and assesses differences in the personal characteristics of vapers in a sample of Norwegian adolescents.

**Aims and Methods:**

Data came from a nationwide longitudinal study of adolescents (n = 2018) conducted in 2017 (T1), 2018 (T2), and 2019 (T3) (mean age: 14.2, 15.0, and 16.2). Using an online questionnaire, we measured vaping with and without nicotine, snus use, smoking, sensation-seeking, conduct problems, and levels of depression.

**Results:**

Past 12-month vaping prevalence was stable (12%, 13%, and 15%). Among adolescents reporting vaping at T1, 66% had used e-cigarettes without nicotine, 22% with nicotine, and 12% were unsure of nicotine content. Individual vaping trajectories were unstable: of nicotine-free vapers, 54% became non-users, while 14% became nicotine vapers from T1 to T2. From T2 to T3, 50% became non-users, while 17% became nicotine vapers. Of nicotine vapers, 39% became non-users from T1 to T2, while 46% became non-users from T2 to T3. Compared to nicotine-free vapers, nicotine vapers had more conduct problems (mean = 3.67 vs 2.17), had more symptoms of depression (mean = 11.38 vs 6.95), and comprised more past 30-day snus users (33% vs 14%) and cigarette users (33% vs 9%).

**Conclusions:**

Adolescent vapers most commonly used e-cigarettes without nicotine, few of these transitioned into nicotine vaping, and a majority became non-users. Nicotine vapers were more likely to use other tobacco products and have more conduct problems and symptoms of depression compared to nicotine-free vapers.

**Implications:**

Reporting the prevalence of nicotine-free vaping is critical for assessing nicotine exposure and subsequent risks of nicotine addiction. This multi-cohort longitudinal study showed that use of nicotine-free e-cigarettes is common among young vapers in Norway. Adolescents’ vaping patterns—both with and without nicotine—are generally temporal and experimental. Despite the majority of nicotine vapers becoming non-users, they are characterized by having more conduct problems and poorer mental health, and they more often used other tobacco products.

## Introduction

The increasing use of electronic cigarettes (e-cigarettes) or vaping devices among young people, in the United States, in particular,^1,2^ has raised concerns about prolonged nicotine addiction in generations growing up in the post-smoking era.^2,3^ Although e-cigarettes may or may not contain nicotine—an important distinction when examining nicotine addiction, studies differentiating between vaping with and without nicotine are rare, and the transition between the two has received little attention. In this study, we examine the user characteristics and stability of vaping with and without nicotine in a sample of Norwegian adolescents assessed at three timepoints in the period from 2017 to 2019.

In Norway, 2% of 15- to 16-year-olds reported daily use of e-cigarettes in 2019, 10% reported use in the last 30 days, while 30% reported having tried.^4^ However, the degree to which these adolescents used e-cigarettes with nicotine is not known. This is also relevant from a regulatory standpoint because the sale of e-liquids that contain nicotine is currently banned. This makes the Norwegian regulatory context similar to the Australian, but different from that of the United States and the United Kingdom.^5^ In Norway, adults are permitted to import e-liquids containing nicotine limited in amount of up to 3 months of personal use, and the majority of users purchase devices and e-liquids online.^6^ In contrast, snus (moist smokeless tobacco) can be legally purchased by persons over the age of 18. Snus is currently the most popular nicotine product among Norwegian adolescents, and 4% of 15- to 16-year-olds reported daily use of snus in 2019, while 2% reported smoking daily.^4^

E-cigarettes have contributed to considerable change in the nicotine and tobacco market in the past decade. In the United States, e-cigarettes are now the most used tobacco product among young people.^1^ The more recent pod-versions, in particular, are found to appeal to young people,^7^ including nonsmokers.^8^ Past 30-day vaping among US high school students increased from 12% in 2017 to 21% in 2018.^9^ In the United Kingdom, 23% of 11- to 18-year-olds reported having tried e-cigarettes. Regular use was more rare, and only 1.6% reported vaping more than once a week.^10^ Correspondingly, a recent qualitative, longitudinal study among Norwegian adolescents portrayed vaping as a time-limited practice. Nicotine vaping was more common and was described as more attractive, compared to nicotine-free vaping, indicating that the unstable user patterns could be attributed to the absence of nicotine addiction.^11^

E-cigarettes have been marketed as nicotine delivery devices,^12^ and the majority of adolescents who report use of e-cigarettes also have experience of other tobacco products.^4,13^ However, many adolescents report that they use nicotine-free e-cigarettes, and alternative user practices have emerged whereby the intake of nicotine is not necessarily the main motivation.^9,14,15^ Examples include vaping to perform tricks,^16^ to satisfy curiosity, to experience flavors, to conform to peer influences,^17–19^ or to administer cannabis.^20,21^

Several longitudinal studies have investigated vaping among young people and the risk of subsequent uptake of combustible tobacco.^22,23^ Several studies have also observed strong associations between the combined use of several tobacco products and e-cigarettes.^24–27^ Older in contrast to younger adolescents, and boys in contrast to girls, are found to be more frequent users of multiple nicotine products.^28–30^

However, only a few longitudinal studies have contrasted transitions in vaping patterns between adolescents using nicotine-free and nicotine e-cigarettes. Treur et al found that experimenting with e-cigarettes both with (12.3%) and without nicotine (27.6%) was widespread among Dutch young people, while regular use was less common. Only 2.5% of the nicotine vapers and 2.6% of the nicotine-free vapers reported past-month use. Notably, past-month vaping was more common among nicotine vapers (9.3%) compared to nicotine-free vapers (4.8%).^25^ Correspondingly, a 6-month follow-up study of vaping among high school students in the United States found that adolescents who vaped with high concentrations of nicotine also reported higher user frequency and intensity of both smoking and vaping.^31^ Similarly, a 2-year longitudinal study in Finland found that experimentation with nicotine vaping, in contrast to nicotine-free vaping, increased the risk of daily nicotine vaping.^14^

In order to fully understand adolescent vaping, more knowledge is needed about how adolescents transition between different types of e-cigarettes, such as from nicotine-free vaping to nicotine vaping. It is also important to examine whether e-cigarettes are used in combination with other nicotine products, such as cigarettes and snus. Finally, there is a need for more knowledge about user characteristics.^23^ For instance, it could be the case that adolescents who vape with nicotine are more sensation-seeking or have more internalized or externalized problems than non-vapers or nicotine-free vapers. Knowledge about such potential risk factors may be important for targeted intervention or prevention efforts.

Against this backdrop, the aims of this longitudinal study were: (1) to identify the prevalence of e-cigarette use, both with and without nicotine, among adolescents aged 14 to 16 in each year from 2017 to 2019; (2) to map transitions between the use of nicotine e-cigarettes, nicotine-free e-cigarettes and nonuse as the participants grow older from 2017; and (3) to determine if these user groups differ in terms of snus and cigarette use, gender, age, sensation-seeking, conduct problems, and symptoms of depression, both cross-sectionally and prospectively.

## Methods

### Data

We analyzed data from the MyLife longitudinal study on adolescent substance use in Norway. Details about the study’s design and recruitment are available in the MyLife Cohort Profile.^32^ The goal of the sampling strategy was to attain a nationwide heterogeneous sample on the criteria of geographical area, urban and rural locations, and standard of living. In total, 42 lower secondary schools were invited to participate. Nine schools declined, leaving 33 schools with a total of 6951 lower secondary pupils. Parents were asked to consent to study participation, and parental consent forms were returned for 4195 students, 3512 of which contained parental consent. The project was approved by the Norwegian Data Protection Authority (reference no.: 15/01495) following ethical evaluation by the National Committee for Research Ethics in the Social Sciences and the Humanities (reference no.: 2016/137).

At the first round of data collection (T1, autumn 2017), a 30-minute online questionnaire was administered by school staff during school hours for students in lower secondary school. Students who had graduated from lower secondary school (10th grade) were contacted individually and completed the questionnaire in their own time. Of the 3512 eligible students, 2975 (85%) completed the questionnaire. All eligible students were invited to follow-ups (T2 and T3, autumn 2018 and 2019). A total of 2857 students (81%) completed the questionnaire at T2, and 2651 (75%) at T3. In the current study, the analysis was restricted to participants who had completed all three assessments and had provided answers to all relevant questions at all three timepoints—in total 2018 adolescents. Compared to the full sample at T1 (n = 2975), the 957 who were not available for follow-up comprised more males (OR = 1.44, *p* < .001), had a lower average symptoms of depression (*b* = −0.55, *p* = .010), and were more likely to have tried cigarettes (OR = 1.60, *p* = .004), but they did not differ from the study sample in terms of age, sensation-seeking, conduct problems, and snus use.

### Measures

Vaping at T1 was measured with the question: “Have you ever tried e-cigarettes?” At T2 and T3, vaping was measured with the question: “Have you used e-cigarettes in the last 12 months?” Possible responses at all timepoints were “Yes” and “No.” Respondents who answered “Yes” received a follow-up question: “When was the last time you used e-cigarettes?” Response options were “Today,” “Not today, but in the last 30 days,” “Not in the last 30 days, but in the last 12 months,” and “More than 12 months ago.” This information was used to define those who were past 12-month vapers at T1. In addition, these four response options were collapsed into a dichotomous yes/no variable measuring vaping in the last 30 days. Smoking and snus use were measured with identical questions, and two variables measuring past 30-day use were created for both smoking and snus use.

Those who answered “Yes” for past 12-month vaping were asked: “Was it with or without nicotine?” Response options were “With nicotine,” “Without nicotine,” and “Don’t know.” Respondents could choose more than one option. Those who indicated “With nicotine,” or both “With nicotine” and “Without nicotine,” comprised the “With nicotine” category (coded 1). Respondents who only indicated “Without nicotine” comprised the “Without nicotine” category (coded 2). Those who only indicated “Don’t know” comprised the “Unsure of nicotine content” category (coded 3). Those who indicated nonuse comprised a “Non-users” category (coded 0).

Sensation-seeking was measured with the 4-item Brief Sensation Seeking Scale.^33,34^ One example item was: “I like to do frightening things.” Responses were structured using a 5-point Likert-type scale, ranging from “Completely disagree” to “Completely agree.” Cronbach’s alpha was 0.79.

Symptoms of depression were measured with the 9-item Severity Measure for Depression (child age 11–17 years) included in the Patient Health Questionnaire.^35^ The reference period was the previous 2 weeks. An example item is: “Feeling down, depressed, irritable, or hopeless.” Responses were structured using a 4-point scale, ranging from “Not at all” (coded 0) to “Nearly every day” (coded 3). The sum of the 9-item scores, ranging from 0 to 27, was used in the analysis. Cronbach’s alpha was 0.90.

Behavioral problems involving misconduct were measured by seven items adopted from the Young in Norway Study.^36^ The items assess the frequency of destruction of property, lying, fighting, sneaking out of home, stealing, being loud and belligerent, and bullying others in the past 12 months. Responses were structured using a 4-point scale, ranging from “Never” (coded 0) to “5 or more times” (coded 3). The sum of item scores comprised the conduct problems index.

### Analysis

Data analysis was conducted using Stata, version 15 (Stata/SE for 15.0 for Windows, 2017). Transitions of e-cigarette use were visualized in a Sankey diagram using the SankeyMATIC tool^37^ based on Supplementary Appendix Table 1. Cohen’s kappa was used to assess the stability of the distributions of individuals over the four vaping categories.^38^ The T1 distribution was successively compared with the T2 and T3 distributions.

Individual characteristics of non-vapers and vapers at baseline (T1) are presented as percentages (for the categorical variables gender, cigarette smoking, and snus use) and means (for the continuous variables age, sensation-seeking, conduct problems, and depression) with 95% confidence intervals.

To examine the possibility that differences between user groups were a consequence of past vaping, we also compared characteristics of respondents who were non-users at all three timepoints with non-users at T1 who later used e-cigarettes *with* nicotine and *without* nicotine at T2 and/or T3, using the same approach. For both sets of analyses, those who were unsure of e-cigarette nicotine content, and those who transitioned into use of ecigarettes without knowledge of nicotine content after T1 were excluded because of low cell count.

## Results

The cross-sectional distributions of past 12-month vapers and non-vapers at T1, T2, and T3 and 95% confidence intervals (95% CI) are shown in Table 1. At T1, 233 of 2018 (11.5%) reported past 12-month vaping, irrespective of nicotine content category. Among the vapers, 66.1% used e-cigarettes without nicotine, 22.3% with nicotine, and 11.6% were unsure of nicotine content. As the cohort grew older, the percentage that had tried vaping during the last 12 months remained at about the same level: 253 of 2018 (12.5%) at T2 and 292 of 2018 (14.5%) at T3.

**Table 1.** Prevalence of Past 12-month Vaping for the Study Sample When Assessed in 2017, 2018, and 2019 (n = 2018). Percentages and 95% Confidence Intervals

	T1 (mean age 14.2)		T2 (mean age 15.2)		T3 (mean age 16.2)	
	n	% (95% CI)	N	% (95% CI)	n	% (95% CI)
Non-users	1785	88.4 (87.0–89.8)	1765	87.5 (85.9–88.8)	1726	85.5 (83.9–87.0)
Use with nicotine	52	2.6 (2.0–3.4)	96	4.8 (3.9–5.8)	147	7.3 (6.2–8.5)
Use without nicotine	154	7.6 (6.6–8.9)	138	6.8 (5.8–8.0)	123	6.1 (5.1–7.2)
Unsure of nicotine content	27	1.3 (0.9–1.9)	19	0.9 (0.6–1.5)	22	1.1 (0.7–1.7)
Total	2018	100	2018	100	2018	100

At T1, the group that used *nicotine-free* e-cigarettes was three times larger than the group that used nicotine e-cigarettes (n = 154, 7.6% vs n = 52, 2.6%). Over time, the share of adolescents using nicotine e-cigarettes increased, while the nicotine-free group decreased, and they became gradually more similar in size at T2 (n = 138, 6.8% vs n = 96, 4.8%) and T3 (n = 123, 6.1% vs n = 147, 7.3%). Few vapers reported not knowing the nicotine content: 27 persons (1.3%) at T1, 19 (0.9%) at T2, and 22 (1.1%) at T3.

Current vaping was less common, only 3.7%, 5.1%, and 5.6% of the sample reported past 30-day vaping at T1, T2, and T3, respectively (not shown in a table). However, the share of current vapers was vastly different between vaping groups. At T1, 50.0% of nicotine vapers reported past 30-day vaping. Among the nicotine-free vapers, the corresponding figure was 26.8%.

**Figure 1.**
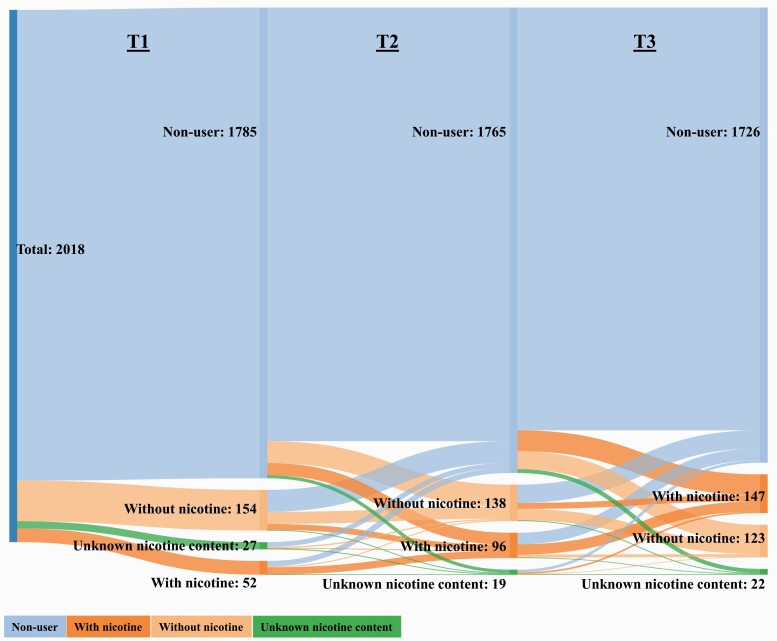
Individual transitions between different e-cigarette use statuses at T1 (2017, mean age 14.2), T2 (2018, mean age 15.0), and T3 (2019, mean age 16.2). n = 2018.

When following the respondents over time (Figure 1), we found that among past 12-month non-vapers at T1 (n = 1785), 92.1% (n = 1644) were still non-users at T2. Among non-vapers at T2 (n = 1765), 90.8% (n = 1603) were non-users at T3.

Among non-users at T1 (n = 1785), 2.6% (n = 46) used nicotine e-cigarettes at T2, and 4.5% (n = 79) of non-users at T2 (n = 1765) had used nicotine e-cigarettes at T3; that is, only a small proportion started vaping with nicotine during the course of the study. The corresponding percentages for non-vaping to nicotine-free vaping were 4.6% (n = 83) for T1–T2, and 3.9% (n = 68) for T2–T3.

Adolescents who used nicotine e-cigarettes were more likely to change status compared to non-users. Among nicotine vapers at T1 (n = 52), 51.9% (n = 27) were still nicotine vapers at T2, but 38.5% (n = 20) were non-users at T2. For T2 to T3, the corresponding figures were 40.6% (n = 39) and 45.8% (n = 44).

Adolescents who used nicotine-free e-cigarettes were more likely to change status compared to nicotine vapers. Among nicotine-free vapers at T1 (n = 154), only 29.9% (n = 46) remained in this category at T2. The corresponding percentage for T2 to T3 was 31.2% (n = 43). One in seven nicotine-free vapers (14.3%, n = 22) transitioned to nicotine vaping from T1 to T2, and one in six nicotine-free vapers at T2 (16.7%, n = 23) transitioned to nicotine vaping from T2 to T3. In contrast, 53.9% (n = 83) of nicotine-free vapers at T1 were non-users at T2, and 50.0% (n = 69) of nicotine-free vapers at T2 were non-users at T3. Therefore, less than 1 in 10 changed status from nicotine-free vapers to nicotine vapers, and the majority changed status to non-users.

The assessment of stability of the distributions of individuals over the four vaping categories using Cohen’s kappa resulted in a κ-value of 0.33 (*p* < .001) for the T1 vs T2, and κ = 0.33 (*p* < .001) for the T2 vs T3 vaping categories distributions, which indicates fair overall stability. For the T1 vs T3 distributions, Cohen’s kappa (κ = 0.13, *p* < .001) suggests only slight overall stability.

Table 2 presents cross-sectional descriptive statistics for non-users, nicotine-free vapers, and nicotine vapers at T1. Compared to non-vapers, vapers were older and had higher scores for sensation-seeking, conduct problems, and depression (all measured at T1). Vapers were also more likely to have used snus and cigarettes in the previous year. There were also notable differences between nicotine and nicotine-free vapers. Nicotine vapers had higher scores for conduct problems and depression and comprised more snus users and smokers.

**Table 2.** Descriptive Statistics for Past 12-month Vaping Groups at Baseline (T1). Percentages and Means With 95% Confidence Intervals

		Non-users at T1 (n = 1785)	Users of e-cigarettes with nicotine at T1 (n = 52)	Users of e-cigarettes without nicotine at T1 (n = 154)
	Range	%/Mean (95% CI)	%/Mean (95% CI)	%/Mean (95% CI)
Variables measured at T1				
Gender (male)	0–1	39.4 (37.2–41.7)	50.0 (36.7–63.3)	57.8 (49.9–65.3)
Age	12–16	14.12 (14.08–14.16)	14.63 (14.43–14.83)	14.53 (14.40–14.65)
Sensation-seeking	1–5	2.85 (2.81–2.90)	3.73 (3.45–4.00)	3.48 (3.34–3.63)
Conduct problems	0–21	0.61 (0.56–0.67)	3.67 (2.62–4.71)	2.17 (1.72–2.62)
Depression	0–27	5.34 (5.09–5.58)	11.38 (9.29–13.48)	6.95 (6.02–7.88)
Snus				
Used in the last 12 months	0–1	2.7 (2.0–3.6)	57.7 (44.0–70.3)	23.4 (17.4–30.7)
Used in the last 30 days	0–1	1.1 (0.7–1.7)	32.7 (21.4–46.4)	13.6 (9.1–20.0)
Cigarettes				
Used in the last 12 months	0–1	1.9 (1.4–2.7)	59.6 (45.9–72.0)	16.2 (11.2–22.9)
Used in the last 30 days	0–1	0.7 (0.4–1.3)	32.7 (21.4–46.4)	9.1 (5.5–14.8)

Table 3 presents descriptive statistics at T1 for adolescents who were non-users at T1, T2, and T3, and adolescents who did not vape at T1 but later reported nicotine or nicotine-free vaping at T2 or T3. Compared to persistent non-users, non-users who had vaped *with* nicotine after T1 were more sensation-seeking, had more conduct problems and symptoms of depression, and comprised more snus and cigarette users. Adolescents who were non-users at T1 but had vaped *without* nicotine after T1 were more similar to persistent non-users, but were more sensation-seeking and had more conduct problems.

**Table 3.** Descriptive Statistics for Persistent Non-Users^a^ and Non-Users at Baseline (T1) Who Later Initiated Vaping With or Without Nicotine. Percentages and Means With 95% Confidence Intervals

	Non-users at T1, T2, and T3 (n = 1501)	Used e-cigarettes with nicotine at T2 and/or T3 (n = 128)	Used e-cigarettes without nicotine at T2 and/or T3 (n = 133)
	%/Mean (95% CI)	%/Mean (95% CI)	%/Mean (95% CI)
Variables measured at T1			
Gender (male)	38.4 (35.9–40.9)	40.6 (32.5–49.3)	51.1 (42.7–59.5)
Age	14.13 (14.08–14.17)	14.17 (14.02–14.32)	14.02 (13.89–14.16)
Sensation-seeking	2.78 (2.73–2.84)	3.29 (3.09–4.50)	3.16 (2.99–3.33)
Conduct problems	0.51 (0.46–0.57)	1.33 (1.01–1.65)	0.92 (0.66–1.19)
Depression	5.14 (4.88–5.40)	7.01 (6.01–8.00)	5.71 (4.81–6.62)
Snus			
Used in the last 12 months	1.7 (1.2–2.5)	11.7 (7.2–18.5)	3.8 (1.6–8.7)
Used in the last 30 days	0.1 (0.0–1.1)	5.5 (2.6–11.0)	*1.5* (*0.4–5.8*)
Cigarettes			
Used in the last 12 months	1.2 (0.8–1.9)	6.3 (3.2–12.0)	3.8 (1.6–8.7)
Used in the last 30 days^b^	*0.3* (*0.1–0.7*)	4.7 (2.1–10.0)	*1.5* (*0.4–5.8*)

^a^E-cigarette users at T1 are not included.

^b^Estimates in italics are based on fewer than five observations.

## Discussion

This study is one of few longitudinal studies that distinguish between adolescents’ vaping with and without nicotine. Approximately 12% of adolescents in this nationwide sample had tried e-cigarettes in the previous year at baseline. Of these, almost two-thirds reported exclusively nicotine-free vaping. Although the overall vaping prevalence only increased slightly between 2017 and 2019, we identified an increase in nicotine vaping, so that nicotine vaping became more popular with increasing age, whereas nicotine-free vaping became less popular. At each timepoint, about one-third of the vapers reported past 30-day vaping, so the majority cannot therefore be considered current vapers. However, current vaping was much more common among nicotine vapers compared to nicotine-free vapers.

In contrast to the overall prevalence, the individual-level vaping patterns were unstable. Large proportions transitioned between e-cigarette use groups (non-use, with and without nicotine). Although there was less stability among nicotine-free vapers compared to nicotine vapers, most adolescents who vaped, including with nicotine, were non-users at a later point in time. Therefore, our findings imply vaping patterns among adolescents marked by experimentation.

In terms of user group characteristics, nicotine vapers differed from the non-users and nicotine-free vapers at baseline. The nicotine vapers had more conduct problems and poorer mental health and were more often users of other tobacco products.

In this study, we identified widespread and infrequent use of nicotine-free e-cigarettes among vapers at baseline, which decreased in proportion with increasing age. This resonates with findings from a Norwegian qualitative study of adolescents’ vaping, where nicotine-free vaping was devalued in status from representing novelty and mild opposition to being seen as uninteresting and childish over a 4-year period.^11^ The increase in nicotine vaping as the cohort grew older corresponds with findings from the United States.^9^ Further echoing findings from the United States and Europe, nicotine vaping in our study was also associated with more frequent use (past 30 days) compared to nicotine-free vaping.^14,15,25,39,40^ Therefore, the increase in nicotine vaping can pose greater concern than nicotine-free vaping, because it is associated with more frequent use and increased likelihood of a subsequent nicotine addiction.^2,9,14^

Despite the increase in nicotine vaping in the overall sample, we found that most vapers changed user status during the study period. This lack of stability indicates that adolescent vaping practices are marked by experimentation.^10,41^ This could be a result of adolescents’ nicotine-free vaping and the associated lower likelihood of addiction. However, the unstable use patterns among adolescents who used nicotine e-cigarettes suggest important factors other than nicotine addiction. Adolescence is a particularly critical period in human development, especially with regards to substance use, when young people become increasingly aware of potential benefits and less convinced of the associated risks.^42^ In contrast to adult vapers who report using e-cigarettes as a smoking cessation aid,^43,44^ alternative motives for vaping have been observed among young people, including curiosity, flavors, performance aspects, perceived coolness, novelty, peer influences, or as mechanisms of misbehavior.^11,17,19^

The common use of nicotine-free e-cigarettes and the experimentation rather than stable and regular use found in our study can also be interpreted in the light of exposure and accessibility. Despite the sale to adolescents of tobacco products and e-cigarettes being prohibited in most parts of the world,^23^ the current ban on nicotine e-cigarettes in domestic shops in Norway may further have limited the respondents’ exposure to vaping, and particularly their access to the latest category of nicotine-flavored pod-devices found to appeal to young people in the United States.^7^

### Characteristics of Vaping Groups

There are several other important explanatory factors to be considered when investigating adolescents’ nicotine-free and nicotine vaping patterns. In our study, we focused on the use of other tobacco products, and known predictors of smoking such as sensation-seeking, deviant behavior, and poor mental health.

One key finding was that, despite frequent transitions between vaping groups, and nonuse during the study period, adolescent vapers still differed from non-users in several ways at T1. As most studies report, vaping was more common among older compared to younger adolescents,^9,45^ more common in boys than in girls,^28,30,45^ and more common among those who used other nicotine products.^23,28,40^ In line with a recent study from the United States,^46^ vaping adolescents were also more sensation-seeking, which could indicate lower self-control.^47^ They also had more conduct problems, and higher average levels of depression compared to non-users. Moreover, at T1, nicotine vapers had poorer mental health compared to nicotine-free vapers, which is in line with the identified higher prevalence of depression among regular vapers.^48^ They also had more conduct problems and higher likelihood of smoking and snus use compared to nicotine-free vapers.

Importantly, the non-users at T1 who later reported nicotine vaping (T2/T3) also had higher levels of depression and more conduct problems, were more sensation-seeking, and were more likely to use cigarettes and snus regularly compared to persistent non-users. While non-users who later reported nicotine-free vaping only were more sensation-seeking and had more conduct problems compared to the persistent non-user group. As suggested by Chan et al, underlying characteristics can thus seem to make some adolescents more likely to engage in practices such as vaping and tobacco use.^23^ Several of these characteristics among vapers, and in particular among the nicotine vapers, have previously been identified as risk factors of smoking. This may indicate common liabilities among adolescents who use e-cigarettes and cigarettes.^23,49^

### Implications

Our findings imply two qualitatively different vaping patterns among adolescents: the use of nicotine-free e-cigarettes seems to indicate more innocent experimentation,^11^ while vaping with nicotine can be associated with more problematic behaviors, such as more frequent vaping, combined use of other tobacco products, and mental health issues.^15,25,39^ Knowledge of such differences is crucial for targeting prevention and intervention. Future research should include monitoring the use of nicotine in adolescents’ vaping patterns.

Persistent monitoring of nicotine-free and nicotine vaping will be particularly relevant in Norway, due to the expected implementation of the Tobacco Product Directive (TPD) in 2022, which will make nicotine e-cigarettes available in domestic outlets. Increased availability may increase adolescents’ nicotine vaping.^9^ In turn, changes in accessibility and exposure to e-cigarettes might require new strategies to prevent increased nicotine use in this age segment.

### Strengths and Limitations

A strength of this study was the use of longitudinal data from a sizeable nationwide study. This made it possible to identify different individual-level use patterns over the course of 2 years. There are also limitations. Firstly, the aim of the sampling strategy was to recruit a nationwide heterogeneous sample of adolescents. The sample was therefore not randomly drawn from the whole population of adolescents. Secondly, because parental consent was a requirement for participation, a considerable number or potential respondents were not reached. Therefore, we have to show caution in generalizing our results relative to the general population of Norwegian adolescents. Thirdly, only a subsample of those included at T1 were available for our analyses at T2 or T3, and those not included comprised more boys, had a lower level of depression, and more often used cigarettes at T1. How this may have affected the results is difficult to ascertain. Fourthly, all measures were based on self-reports, which can lead to known biases, such as selective recall and socially desirable responses, especially among younger participants. Fifthly, because nicotine vaporizers, pods, and liquids are unavailable among Norwegian vendors under the current regulatory regime, generalization concerning countries with a different product portfolio may be limited. Lastly, due to low prevalence, a relatively small number of adolescents in the sample had vaped with nicotine at T1. This limited our analysis options and our ability to detect small differences. A considerably larger dataset would have been required to differentiate between use frequency groups (eg, daily, weekly, monthly, and rarely), but this would have allowed detailed mapping of transitions between use frequency groups and detailed examination of risk factors for such transitions. Studies that include repeated measures for samples that include sufficient numbers of adolescent daily vapers (with and without nicotine) should be conducted to provide more detailed knowledge about adolescent vaping.

## Conclusions

In this study, 1 in 9 of the Norwegian adolescents reported past 12-month vaping, and 1 in 25 reported past 30-day vaping at baseline. Of these past year vapers, 7 out of 10 used nicotine-free e-cigarettes, while only 2 in 10 used nicotine e-cigarettes. Further, adolescents’ vaping trajectories, both with and without nicotine, were unstable, which indicates that experimentation does not necessarily lead to stable use patterns. Nonetheless and irrespective of nicotine use, we find that adolescents who use e-cigarettes differ from non-users. Vapers were older, they more often used cigarettes and snus and they had higher scores for sensation-seeking, depression, and conduct problems. In addition, nicotine and nicotine-free vapers differ from one another. Nicotine vapers were more likely to use other tobacco products, and have more conduct problems and higher levels of depression compared to their nicotine-free vaping peers. In summary, these findings highlight the importance of monitoring both nicotine-free and nicotine vaping in future studies on vaping among adolescents.

## Supplementary Material

A Contributorship Form detailing each author’s specific involvement with this content, as well as any supplementary data, are available online at https://academic.oup.com/ntr.

ntab192_suppl_Supplementary_Appendix

ntab192_suppl_Supplementary_Taxonomy-form

## Data Availability

Due to confidentiality agreements, supporting data can currently only be made available to researchers affiliated with the MyLife project. Details of the data and how to request access will become available from Geir Brunborg at the Norwegian Institute of Public Health.
