# Supplementary material for: Adolescents’ Use of Nicotine-Free and Nicotine E-Cigarettes: A Longitudinal Study of Vaping Transitions and Vaper Characteristics
Source: Nicotine Tob Res. 2021 Sep 21;24(3):400–7. doi: 10.1093/ntr/ntab192 (PMC8842395; doi:10.1093/ntr/ntab192)
Supplement: ntab192_suppl_Supplementary_Appendix [file ntab192_suppl_supplementary_appendix.pdf]

Appendix 1: Cross tabulation of respondents with different e-cigarette use statuses at T1 vs T2 and T2 vs T3

| | T1 | | T2 Non-users | | T2 With nicotine | | T2 Without nicotine | | T2 Unsure of nicotine content | |
|---|---|---|---|---|---|---|---|---|---|---|
| | n | % | n | % | n | % | n | % | n | % |
| **Non-users T1** | **1785** | **100.0** | 1644 | 93.4 | 46 | 1.9 | 83 | 4.1 | 12 | 0.6 |
| **With nicotine T1** | **52** | **100.0** | 20 | 43.1 | 27 | 47.7 | 4 | 7.7 | 1 | 1.5 |
| **Without nicotine T1** | **154** | **100.0** | 83 | 56.0 | 22 | 13.5 | 46 | 29.0 | 3 | 1.5 |
| **Unsure of nicotine content T1** | **27** | **100.0** | 18 | 70.0 | 1 | 10.0 | 5 | 12.0 | 3 | 8.0 |
| **Sum** | **2018** | **100.0** | 1765 | 87.5 | 96 | 4.8 | 138 | 6.8 | 19 | 0.9 |
| | **T2** | | **T3 Non-users** | | **T3 With nicotine** | | **T3 Without nicotine** | | **T3 Unsure of nicotine content** | |
| | **n** | **%** | **n** | **%** | **n** | **%** | **n** | **%** | **n** | **%** |
| **Non-users T2** | **1765** | **100.0** | 1603 | 90.8 | 79 | 4.5 | 68 | 3.9 | 15 | 0.8 |
| **With nicotine T2** | **96** | **100.0** | 44 | 45.8 | 39 | 40.6 | 11 | 11.5 | 2 | 2.1 |
| **Without nicotine T2** | **138** | **100.0** | 69 | 50.0 | 23 | 16.7 | 43 | 31.2 | 3 | 2.2 |
| **Unsure of nicotine content T2** | **19** | **100.0** | 10 | 52.6 | 6 | 31.6 | 1 | 5.3 | 2 | 10.5 |
| **Sum** | **2018** | **100.0** | 1726 | 85.5 | 147 | 7.3 | 123 | 6.1 | 22 | 1.1 |
